# Late outcome of surgical radiofrequency ablation for persistent valvular atrial fibrillation in China: a single-center study

**DOI:** 10.1186/s13019-017-0627-z

**Published:** 2017-08-03

**Authors:** Hailong Cao, Yunxing Xue, Qing Zhou, Minggang Yu, Chenbin Tang, Dongjin Wang

**Affiliations:** Department of Thoracic and Cardiovascular Surgery, The Affiliated Drum Tower Hospital of Nanjing University Medical School, 321 Zhongshan RD, Nanjing, 210008 China

**Keywords:** Atrial fibrillation, Surgical radiofrequency ablation, Late outcome, China

## Abstract

**Background:**

Atrial fibrillation (AF) adversely affects surgical outcomes of cardiac valve surgery. Surgical ablation is an effective way to treat valvular AF. The aim of this study was to evaluate the late outcome of surgical radiofrequency ablation and explore the risk factors of AF recurrence in Chinese patients undergoing cardiac valve surgery.

**Methods:**

Three hundred ninety six consecutive patients with persistent valvular AF were enrolled in this study. They underwent concomitant modified Maze IV and were completed follow-ups. Cox survival regression model was used to screen independent risk factors for predicting late recurrence of AF.

**Results:**

During the follow-up period of 28 (6 ~ 61) months, AF recurred in 151 patients (38.1%). In multivariate survival regression, factors predictive of AF late recurrence were AF duration, pre-operative serum B-type natriuretic peptide (BNP), pre-operative heart rate and left atrial diameter (LAD), post-operative atrial tachyarrhythmias and AF at discharge. According to receiver operating characteristic curve analysis, the best cutoff values for AF duration, BNP, heart rate and LAD were 66.5 months, 251 pg/ml, 82 beats/min and 67.9 mm, respectively.

**Conclusions:**

Longer AF duration, larger LAD, higher BNP level and lower heart rate indicated a poor late outcome of surgical radiofrequency ablation in persistent AF patients undergoing cardiac valve surgery. Therefore, indication to radiofrequency ablation should be carefully considered in these patients.

**Electronic supplementary material:**

The online version of this article (doi:10.1186/s13019-017-0627-z) contains supplementary material, which is available to authorized users.

## Background

Atrial fibrillation (AF) is the most common sustained cardiac arrhythmia in patients undergoing cardiac valve surgery, contributing to increased risks of systemic embolization, anticoagulant-related hemorrhage and mortality [1]. The Maze III procedure had been the gold standard in the surgical treatment of AF [2]. But the complexity and complications prevented its widespread application. As the advance of energy medicine, Maze IV procedure based on different kinds of energy was confirmed its effective role in treating concomitant AF [3]. Because radiofrequency ablation was used earliest in clinic, it was more broadly applied worldwide and began to be performed in more and more cardiac centers of China since 2005 [1].

Most randomized trials have demonstrated an excellent conversion rate of the sinus rhythm (SR) with surgical radiofrequency ablation [4], but 20% to 30% of those undergoing the maze operation showed recurrent AF during the follow-up period [5, 6]. In China, particularly, the cause of valvular AF is mostly rheumatic heart disease. These patients commonly have a long history of persistent AF and enlarged left atria, which indicated an advanced degree of atrial structural remodeling [7]. Due to lack authoritative analysis of risk factors for late outcome in this population, there is no standard before cardiac valve surgery to choose which patients should not be performed concomitant radiofrequency ablation. Therefore, we herein aimed to explore clinical and instrumental factors predictive of late recurrence after concomitant radiofrequency ablation in Chinese patients undergoing cardiac valve surgery.

## Methods

### Patient enrollment

We consecutively recruited 510 valvular AF patients with written informed consent admitted to the Affiliated Drum Tower Hospital of Nanjing University Medical School between April 2010 and November 2014. A pre-ablation 7-day Holter and comprehensive transthoracic echocardiographic examination were performed to establish that all patients had persistent valvular AF. The study was conducted according to the Helsinki Declaration and approved by the ethics committee of Nanjing University.

Complete clinical data were collected before discharge. There were 114 patients excluded: 11 cases died in hospital due to low cardiac output syndrome and multiple organ failure; 2 cases died of sudden death after discharge; 2 case died of heart failure after discharge; 8 cases died of trauma or cerebrovascular accident; 7 cases had a history of implanted pacemaker before surgery; 8 cases received pacemaker implantation because of sinus long-intermission rhythm and advanced atrioventricular block. And another 76 cases were lost at follow-up. Therefore, a total of 396 patients had complete follow-ups and clinical information.

### Surgical procedures

All surgical procedures were performed in standard fashion by the same surgical team through median sternotomy. All patients underwent either a mitral valve repair or replacement by cardiopulmonary bypass with a mild hypothermia (32-34 °C), antegrade cold blood cardioplegic arrest, and local hypothermia with ice-slash; cardioplegic solution was readministered every 20 to 30 min. Radiofrequency ablation was performed using a mono-polar radiofrequency device (Cardioblate®; Medtronic, USA). Left and right atrial appendages and the ligament of Marshall were incised routinely. We did vertical right atrial incision between the superior and inferior vena cava in all patients. Maze IV was performed before the other intra-cardaic procedures. The lines performed included isolation of the right and left pulmonary veins firstly and the connective lines between them. Next lines were from isolating line of the left pulmonary vein to the base of left atrial appendage amputation site and to the posterior mitral valve annulus. In the right atrium, the isthmus ablation runs from the inferior caval vein, across the interatrial septum, up to the caudal aspect of the coronary sinus ostium and over to the posterior tricuspid valve annulus. If patients had a huge left atrium, we would add several auxiliary ablation lines across left atrial roof and posterior wall. Epicardial temporary pacing wires were implanted in all patients.

### Post-operative care and follow-up

Heart rhythm was continuously monitored after surgery. Dual-chamber stimulation at 80 bpm through epicardial temporary pacing wires was used in most of patients for the first 48 h post-operatively to avoid severe brady arrhythmias. After that, all patients received amiodarone and metoprolol to stable rhythm except these patients whose heart rate is too slow to tolerate these antiarrhythmic drugs. AF recurrences after surgery were treated by resuming amiodarone infusion and correcting, in the case, electrolyte abnormalities. In patients in whom AF persisted despite optimal medical therapy, at least one attempt of electronic cardioversion was performed before discharge.

Late recurrence was defined as any episode of AF, atrial flutter or atrial tachycardia that lasted greater than 30 s at least 6 months after surgery [8]. AF-free time was calculated from the date of ablation to the date of recurrence or last follow-up 28 (6 ~ 61) months. Patients had scheduled clinical visits. 12-lead electrocardiography and 24-h Holter monitoring were monthly conducted after 6 months within the first year and then chosen every half year after that. Moreover, patients would receive electrocardiography monitoring in local clinics at anytime if they had AF-related symptoms. Within the first 6 months, patients who experienced AF recurrence would receive electronic cardioversion to maintain SR. Late recurrence of AF during the follow-up was considered censored.

### Statistical analysis

For the comparison between the two groups, Student’s t–test (normally distributed) or Mann–Whitney test (non-normally distributed) was used for continuous variables, and χ^2^ test was utilized for categorical variables. Cox survival regression model was used to determine factors predictive of AF late recurrence. Factors for which the univariate analysis gave a *p* value ≤0.1 were included in the multivariate analysis. Hazard ratio (HR) and 95% confidence interval (CI) were calculated. Youden index (Sensitivity + Specificity-1) of receiver-operating characteristic (ROC) curve was calculated to single out the best cutoff values of AF duration, pre-operative B-type natriuretic peptide (BNP), pre-operative heart rate and left atrial diameter (LAD) predicting AF late recurrence. Sensitivity, Specificity and Accuracy were investigated by the Fisher’s exact test. Survival curves for the incidence of AF recurrence by AF duration, pre-operative BNP, pre-operative heart rate and LAD were calculated with the Kaplan-Meier method and a log-rank test was used to assess statistical significance. *p* < 0.05 was considered statistically significant, and all statistical tests were two-sided. The statistical analysis was performed with the GBSTAT statistical analysis package (version 9.0, Dynamic Microsystems, Inc).

## Results

### Patient characteristics

The 396 patients were divided into two groups according to whether AF late recurrence took place or not (SR group *n* = 245, AF group *n* = 151, see Table 1). No significant differences were found in terms of gender, age, the ratio of rheumatic cause, cerebral infarction, pre-operative left ventricular ejection function, combined tricuspid valve annuloplasty and coronary artery bypass grafting, cardiopulmonary bypass duration, aortic clamp time, intensive care unit stay, post-operative using of amiodarone and metoprolol. The AF group had longer AF duration and higher percents of hypertension, left atrial thrombosis and redo-procedure than the SR group. However, the AF group had lower ratio of diabetes and worse New York Heart Association class than the SR group. The pre-operative echocardiographic LAD was larger and pre-operative serum BNP was higher in AF than SR, but heart rate in the AF group were slower than SR group in the admission. Moreover, the AF group experienced a shorter duration of ventilation and higher ratio of AF at discharge and electronic cardioversion within 6 months than the SR group.

**Table 1:** Clinical characteristics of study population

	SR Group *n* = 245	AF Group *n* = 151	P valve
Gender, M/F (n)	132/113	88/63	0.392
Age (yrs)	55.7 ± 10.5	57.3 ± 7.8	0.096
AF duration (mhs)	33.1 ± 25.5	69.0 ± 67.7	<0.001
Rheumatic valvular disease (n)	183	108	0.488
Cerebral infarction (n)	26	12	0.382
Hypertension (n)	31	34	0.010
Diabetes (n)	22	5	0.030
Left atrial thrombosis (n)	29	39	<0.001
NYHA class (I-II/III-IV)	99/146	42/109	0.011
Pre-op BNP (pg/ml)	202.7 ± 124.7	367.5 ± 295.7	<0.001
Pre-op heart rate (bpm)	95 ± 19	75 ± 12	<0.001
Pre-op LAD (mm)	57.0 ± 8.6	61.4 ± 12.0	<0.001
Pre-op LVEF (%)	50.8 ± 6.8	49.5 ± 6.2	0.063
Redo-procedure (n)	7	22	<0.001
Combined tricuspid valve annuloplasty (n)	243	149	0.623
Combined coronary artery bypass grafting (n)	25	8	0.086
Cardiopulmonary bypass duration (min)	168 ± 44	169 ± 44	0.815
Aortic clamp time (min)	133 ± 39	130 ± 40	0.364
Duration of ventilation (hrs)	22.4 ± 19.4	34.5 ± 56.0	0.011
Intensive care unit stay (dys)	4.6 ± 2.1	5.0 ± 2.1	0.058
Post-op atrial tachyarrhythmias (n)	89	129	<0.001
AF at discharge (n)	57	123	<0.001
Post-op amiodarone (n)	79	41	0.284
Post-op metoprolol (n)	151	84	0.237
Electronic cardioversion within 6 months (n)	29	37	0.001

Values are presented as mean ± SD or number of patients

*AF* atrial fibrillation, *BNP* B-type natriuretic peptide, *LAD* left atrial diameter, *LVEF* left ventricular ejection function, *NYHA* New York Heart Association, *Pre-op* pre-operative, *Post-op* post-operative, *SR* sinus rhythm

### Predictors of AF late recurrence

In the Cox univariate regression analysis, significant predictors of AF late recurrence were AF duration, hypertension, left atrial thrombosis, New York Heart Association class, pre-operative serum BNP level, pre-operative heart rate and LAD, pre-operative left ventricular ejection fraction, redo-procedure, post-operative atrial tachyarrhythmias, AF at discharge and post-operative amiodarone.

In the multivariate model, AF duration, pre-operative serum BNP level, pre-operative heart rate and LAD, post-operative atrial tachyarrhythmias and AF at discharge remained statistically significant predictors of AF late recurrence (Table 2).

**Table 2:** Predictors of AF recurrence in Cox multivariate survival regression analysis

Variables	β	SEM	HR	95% CI	*P* Value
AF duration	0.322	0.131	2.762	1.831 ~ 3.664	<0.001
Pre-op BNP	0.141	0.039	2.964	1.943 ~ 4.177	<0.001
Pre-op heart rate	−0.320	0.006	0.668	0.657 ~ 0.680	<0.001
Pre-op LAD	0.330	0.062	3.017	2.872 ~ 3.255	<0.001
Post-op atrial tachyarrhythmias	0.614	0.263	1.823	1.184 ~ 3.643	0.012
AF at discharge	0.857	0.321	2.355	1.255 ~ 4.419	0.008

*β* regression coefficient, *SEM* standard error of the mean, *HR* hazard ratio, *CI* confidence interval

### Predictive values of AF duration, pre-operative BNP, pre-operative heart rate and LAD

According to the ROC curve analysis, the best threshold values of AF duration, pre-operative serum BNP level, pre-operative heart rate and LAD were 66.5 months (39.7% sensitivity, 89.0% specificity and 55.3% accuracy), 251 pg/ml (60.3% sensitivity, 71.4% specificity and 67.2% accuracy), 82 beats/min (75.1% sensitivity, 81.5% specificity and 75.8% accuracy) and 67.9 mm (31.8% sensitivity, 91.4% specificity and 68.7% accuracy), respectively (Table 3).

**Table 3:** Predictive values for AF recurrence by receiver-operating characteristic curve

Variables	Best Cutoff Values	AUC (95% CI)	Sensitivity (%)	Specificity (%)	Accuracy (%)
AF duration	66.5 mhs	0.653 (0.593 ~ 0.712)	39.7	89.0	55.3
Pre-op BNP	251 pg/ml	0.691 (0.636 ~ 0.746)	60.3	71.4	67.2
Pre-op heart rate	82 bpm	0.842 (0.802 ~ 0.882)	75.1	81.5	75.8
Pre-op LAD	67.9 mm	0.600 (0.540 ~ 0.660)	31.8	91.4	68.7

Best Cut-off value is equal to the biggest Youden index

Youden index = Sensitivity + Specificity-1. AUC, area under the curve

**Fig. 1:**
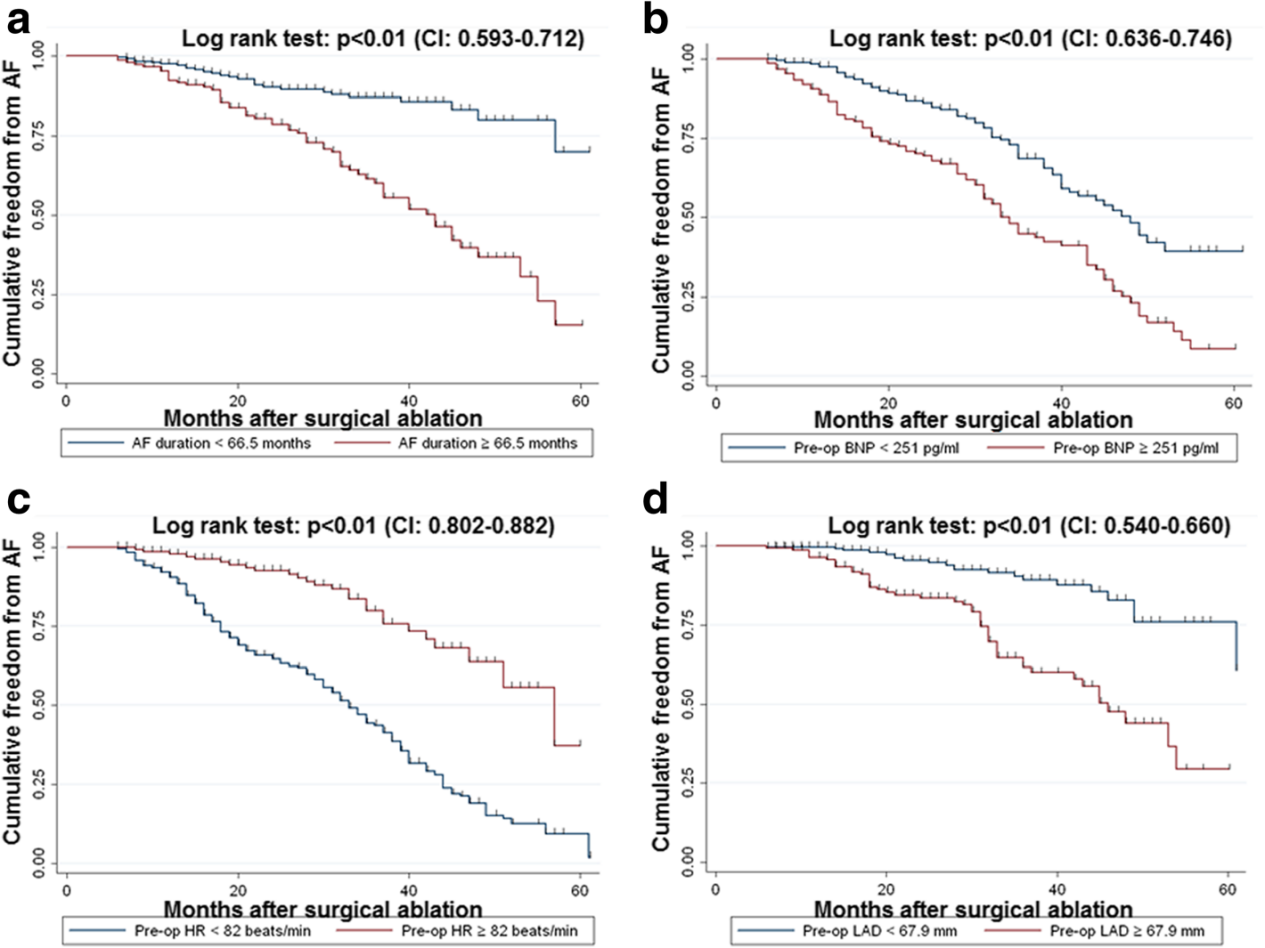
Kaplan-Meier survival curves showing freedom from AF recurrence by (**a**) AF duration, (**b**) Pre-op B-type natriuretic peptide, (**c**) Pre-op heart rate and (**d**) Pre-op *left* atrial diameter. CI, confidence intervals

In addition, Kaplan-Meier survival estimates showed that patients with AF duration ≥66.5 months, pre-operative serum BNP level ≥ 251 pg/ml and pre-operative LAD ≥67.9 mm had higher proportions of AF late recurrence than patients with AF duration <66.5 months (Fig. 1a), pre-operative serum BNP level < 251 pg/ml (Fig. 1b) and pre-operative LAD <67.9 mm (Fig. 1d), respectively (*p* < 0.001). However, Patients with pre-operative heart rate > 82 bpm had a much lower proportion of AF late recurrence than patients with pre-operative heart rate ≤ 82 bpm (*p* < 0.001) (Fig. 1c).

## Discussion

Surgical ablation of valvular AF by means of radiofrequency is a well-accepted strategy to treat concomitant AF. Bando et al. in a study of 812 patients, who had undergone mitral valve replacement and Maze procedure, reported a 97% 8-year freedom from stroke in presence of restored SR as compared with 89% in presence of recurrent AF [9]. Similar data were observed in other studies [6, 10]. Moreover, the possibility to restore the SR can increase the chance to perform valve replacement with a biological prosthesis to avoid lifelong therapy with warfarin, particularly in old patients, in which it is preferable to reduce the higher risk of bleeding [11]. Therefore, survival, functional status and life quality will be much better in patients in SR after Maze procedure.

Late outcomes for our group after radiofrequency ablation were encouraging. Our 61.8% success rate at an average follow-up time of 28 months compares similarly with other literature reports [5]. Although selected single-center studies have shown rates of post-ablation freedom from AF of 80% or more, 1-year estimates of approximately 70% are more typical [12, 13]. One reason is the clinical characteristics of our patients. The patients with pre-operative giant left atrium (>65 mm) comprised a big proportion of 94 patients (23.7%) in our study and most of the patients in our study experienced long AF duration. Another reason might be our surgical technique, in which the maze around the pulmonary veins and mitral annulus might be not narrow enough to interrupt reentrant circuits when the left atrium was a giant one. Therefore, additional lines should be performed to leave only a 2 to 3 cm gap to prevent the potential reentrant circuits in these patients [14].

In present investigation, we found a lower sinus conversion rate was associated with a longer AF duration and a larger LAD. Of particular interest, left atrial size and AF duration are significantly and mutually related to each other. Many clinical investigations have recognized left atrial dilatation as a cause of AF [15]. Left atrial dilatation, on the other hand, may also be a consequence of AF [16]. Therefore, they together aggravate atrial fibrosis and remodeling, making patients more susceptible to experience a recurrence of AF. Melo et al. believed that AF duration >4 years and LAD >5.5 cm are the boundaries to predict the efficacy of surgical treatment via the maze procedure [17]. The data from Zongtao et al. showed that AF duration ≥7 years and LAD ≥58 mm increased the AF recurrence rate by more than twofold [18]. Herein, we found that AF duration ≥66.5 months and pre-operative LAD ≥67.9 mm indicated a significantly higher ratio of AF recurrence. It has slightly expanded the previous indications for the efficacy of the surgical maze procedure.

Baseline serum BNP had been previously found predicting short-term recurrence in patients undergoing catheter ablation of AF [19]. Herein, we also identified that pre-operative serum BNP level ≥ 251 pg/ml was associated with a much lower post-ablation freedom from AF. The increased atrial stretch associated with increased left atrial volume can be responsible for the elevated levels of plasma BNP, because BNP is released primarily from the atria [20]. Increased wall stress by the diastolic dysfunction itself that frequently coexists with AF may also induce the release of BNP from the ventricle [21]. Such complex and various mechanisms regarding to BNP release may make its levels heterogeneous among individuals with AF, consequently making its clinical role unclear. In our patients with left atrial enlargement and long-standing AF, BNP level was bound to be more elevated, because they were significantly correlated with each other. However, due to lacking consecutive monitoring of BNP after surgery, the role of post-operative BNP for predicting SR stabilization is unknown.

Herein, we also found that early post-operative atrial tachyarrhythmias and AF at discharge was another two predictors for late recurrence of AF. It was in agreement with the results of previous reports [22, 23]. These findings suggested that part of the atrial arrhythmogenic substrate for early and late recurrence of AF might be similar. Arial remodeling includes structural and electrophysiological remodeling, most notably changes in substrate refractory period, amplitude and duration of action potential, and conduction velocity. These modifications appear to establish an environment that facilitates multiple reentry activity [24]. Experimental animal model studies have shown that when AF is maintained over time, it tends to recur more frequently and finally becomes sustained [24]. The progressive self-perpetuating nature of this arrhythmia, aptly described as “AF begets AF”, occurs simultaneously with atrial substrate remodeling [24]. Therefore, the both parameters found in this study must be associated with cardiomyopathy in atria and appear likely to reflect “resistance” to surgical ablation of persistent valvular AF.

Moreover, pre-operative lower heart rate (≤82 bpm) was found for the first time as a risk factor for predicting late recurrence of AF in our population. Heart rate was collected from the average ventricular rate of a preoperative 7-day Holter. The possible mechanism is that the function of sinus node is degrading in patients with chronic AF. The effective refractory period, which is associated with the formation and maintenance of atrial reentrant cycle, is prolonging accompanied with the advance of degraded sinus node [25]. Thus, based on this deduction, it is difficult to eliminate chronic AF in these patients with pre-operative low heart rate. However, the detailed mechanisms remain to be elucidated in further.

### Limitations

Several limitations of this study require consideration. First, the nonrandomized design may have affected the results because of unmeasured confounders, procedure bias or detection bias, despite the use of rigorous statistical adjustment. Second, because of the initial deficiency of mono-polar radiofrequency device, defined as the lack of intra-operative lesion integrity assessment, it may have left some patients with gap lesions, ultimately affecting outcome. Third, all procedures were performed in a single institution. A related potential bias may result from the reliance on self reporting. So a multi-center study with a large population is warrant to confirm our findings. Moreover, observed freedom from AF varies with the rigor of rhythm assessment. As compared with continuous long-term monitoring, spot electrocardiographic recordings tend to overestimate success by approximately 12 percentage points [26]. Therefore, a study using an implantable continuous monitor is warranted to improve our finding.

## Conclusions

Our data suggest that indication to surgical treatment of AF should be carefully weighted in patients with long AF duration, pre-operative high serum BNP level, low heart rate and enlarged left atrium. Early post-operative atrial tachyarrhythmias and AF at discharge indicate a worse late outcome of surgical radiofrequency ablation. Thus, identification of these risk factors may improve selection of subjects to obtain long-term results in patients with persistent AF undergoing cardiac valve surgery.

## Additional file

Additional file 1: Original data. (XLS 131 kb)

## Abbreviations

AF: Atrial fibrillation
BNP: B-type natriuretic peptide
CI: Confidence interval
HR: Hazard ratio
LAD: Left atrial diameter
ROC: Receiver-operating characteristic
SR: Sinus rhythm
